# Evaluation of a Portable Handheld Heterochromatic Flicker Photometer in Measuring Macular Pigment Optical Density

**DOI:** 10.3390/diagnostics15040431

**Published:** 2025-02-11

**Authors:** Pinakin Gunvant Davey, Richard B. Rosen, Joshua J. Park, Frank Spors, Dennis L. Gierhart

**Affiliations:** 1 College of Optometry, Western University of Health Sciences, Pomona, CA 91766, USA; fspors@westernu.edu; 2 ZeaVision LLC, Chesterfield, MO 63005, USA; dgierhart@zeavision.com; 3 Department of Ophthalmology, New York Eye and Ear Infirmary of Mount Sinai, Icahn School of Medicine at Mount Sinai, New York, NY 10029, USA; rrosen@nyee.edu; 4 College of Engineering, California Baptist University, Riverside, CA 92504, USA; joshuaparkresearch@gmail.com

**Keywords:** macular pigment optical density, heterochromatic flicker photometry, carotenoids, lutein, zeaxanthin, meso-zeaxanthin, macular degeneration

## Abstract

**Background/Objectives:** Macular pigment optical density (MPOD) is an important clinical biomarker for ocular conditions like macular degeneration, diabetic eye disease, and digital eye strain. Additionally, its measurements can be essential in health assessment for visual function, systemic diseases, and brain health. We aimed to assess the repeatability, agreement, and effects of the learning curve of the new portable handheld heterochromatic flicker photometer, Zx Pro, in measuring MPOD in a wide age range of ocular-healthy adults, compared to the MPOD measurements obtained using the clinically available QuantifEye device. **Methods:** Seventy-six participants performed one practice attempt and two study-related MPOD measurements with the Zx Pro and the QuantifEye. **Results:** The Pearson correlation between the study-related MPOD measurements for Zx Pro and QuantifEye devices was 90% and 85%, respectively. Bland and Altman plots show excellent agreement between the device’s MPOD data, with 95% limits of an agreement being −0.10 to +0.11 du. The mean difference between the practice attempt and the study-related measurements was not statistically significant for Zx Pro but was significant for QuantifEye (Repeated measures ANOVA *p* = 0.325 and *p* = 0.015, respectively). **Conclusions:** The Zx Pro provides excellent repeatable MPOD measurements, has an insignificant learning curve, and is in good agreement with the predicate device.

## 1. Introduction

The dietary xanthophyll carotenoids, lutein and zeaxanthin, along with an isomer of zeaxanthin, meso-zeaxanthin, collectively comprise the macular pigment, which is observed as a central yellow spot in fundus evaluation [1,2]. While not visualizable using ophthalmoscopy or retinal photography, xanthophylls are present in lower amounts throughout the retina and have an integral role in the visual function and maintenance of retinal health [1,2,3,4]. Xanthophylls carotenoids serve as the eye’s inherent antioxidant capacity that protects the eye from harmful radiation, quenching free radicals generated during metabolism and benefitting visual function by optical and non-optical mechanisms [3]. The carotenoids in the human body cannot be synthesized de novo and are obtained by diet. There are indeed various dietary sources for ocular xanthophylls, like spinach, kale, Swiss chard, orange peppers, asparagus, eggs, etc., to name a few [5].

Heterochromatic flicker photometry (HFP) is currently most commonly used technology in the measurement of MPOD, both clinically and in research settings [1,6]. The amount of xanthophyll carotenoids in the retina is measured as MPOD. Although MPOD is known popularly for its association with macular degeneration [7,8,9], MPOD measurements are gaining significant notoriety as an oculo-systemic biomarker [10]. It is now known that dietary intake of carotenoids and sub-optimal MPOD are measurable and modifiable risk factors in numerous conditions like macular degeneration [8,9,11], diabetes [12], and pathologies like glaucoma [13]. The level of MPOD is correlated with visual performance [10,14,15], cognitive performance [16], and, more recently, digital eye strain [17,18] that affects a significant part of the global population [19]. Furthermore, macular pigment levels in the retina are highly correlated to the levels of the carotenoids in the occipital cortex [20], thus making its measurements additionally valuable for systemic and brain health [21]. Additionally, due to its measurement principles, the MPOD measured by the HFP is a direct assessment of the eyes’ ability to absorb blue light [6,22,23,24].

While current commercial HFP devices are easy to use, repeatable, reliable, and able to track changes in diet and intake of nutritional supplements [25], they are marred with being space-occupying desktop units that are not portable and tend to obstruct smooth flow in busy clinical practices that see a significant number of patients daily. Recently, EyePromise/ZeaVision LLC St Louis, MO USA made a portable, handheld HFP device available to improve the clinical usability of this technology. The current study aims to evaluate the new portable device and compare its repeatability, learning curve, and MPOD measurements with the currently commercially available HFP QuantifEye MPS II device (IDE Vision Ltd., Caldicot, UK). We hypothesize that there should be a positive correlation and agreement between the two devices despite the software and hardware differences given these both are based on principles of HFP. The outcome of this study will allow the establishment of clinically valuable protocols. It will also provide helpful information for clinicians and researchers and aid future clinical trials in nutrition, eye care, and systemic physiology.

## 2. Materials and Methods

Participants were evaluated by a single trained optometric physician (PGD) at the Western University of Health Sciences, College of Optometry. The study was approved by the institutional review board at the Western University of Health Sciences, Pomona, CL, USA, and conducted in accordance with the tenets of the Declaration of Helsinki (P20/IRB/060 2022). The institutional review board at the Western University of Health Sciences deemed the study as minimal risk as both devices use light levels that are very safe for the human eye, and no invasive procedures were involved in the study. A campus-wide email and flyers were distributed, and the participants included faculty, staff and students, and their friends and family members. Participants read and signed a consent form in a language that they understood, were adults of at least 18 years of age, and were deemed to have good ocular health during a comprehensive ocular evaluation by an optometric physician in the last year. Participants additionally underwent visual acuity assessments and refractive error estimation during the study. Participants with a history of retinal pathology, glaucoma, or history of surgery that could influence measurements were excluded. This was carried out as the retinal health is compromised in these conditions, and visual function is decreased in individuals with these conditions. All participants had a logMAR visual acuity of +0.01 (20/25) or better.

A total of 76 eyes of 76 participants were included in the study. The mean age of the participants was 33.9 years (standard deviation 11.57; range 23–70 years).

The median spherical refractive error for the study participants was −1.68 D (range +0.50 to −7.00 D). All participants were novice observers who were not experienced in the heterochromatic flicker photometry (HFP) procedure to measure MPOD.

### 2.1. Macular Pigment Optical Density (MPOD) Measurement

The MPOD was assessed using two devices, the QuantifEye MPS II (IDE Vision Ltd., Caldicot, UK) and the Zx Pro device (EyePromise/ZeaVision LLC, Chesterfield, MO, USA). The fundamental principles of these two devices are based on HFP [26]. They both measure the MPOD in the central 1 degree of the macula. The HFP devices measure the MPOD by measuring the retinas’ ability to absorb the blue spectra that are converted to density units. The devices have a slot to insert a trial lens slot to insert patients’ distance refractive error when needed. During the testing, participants were instructed to pay attention to a single target or spot. An individual undergoing the testing for an eye was directed to look at a target, which is a single light source, and asked to maintain the position throughout the test. The fellow eye not being examined was patched to ensure the eye’s steady fixation and avoid binocular rivalry. At this point, the light source illuminates the center of the fovea around 1 degree. The light source was an LED light, the blue wavelength was 465 nm, and the green wavelength was 530 nm.

The HFP is based on the principle that, when the blue and green spectral targets are projected in rapid succession to the retina, the individual experiences the target flickering. Individuals performing the test are instructed to press a trigger whenever they see the target flickering. This flicker is sometimes reported and better understood as shimmer or static. Subsequently, the computer changes to the blue versus green ratio, in which the target appears flickering. The test continues for approximately two minutes with a decreasing blue-to-green ratio. When the retina absorbs the maximum blue light, the individual will not perceive the flicker. The testing continues three more times, where the amount of blue is higher than that absorbed by the fovea, which the individual perceives and then responds to the flicker. These devices’ inbuilt automated software calculates a score ranging from 0 to 1, with 1 being the maximum MPOD score. The MPOD values are adjusted for the age-related yellowing of the lens. Although not applicable to the current study, individuals who have undergone cataract extraction with intraocular lens implantation have clear media, and their age should be recorded at 21 years, where no age-related adjustment is required.

The fundamental principles of these two devices are based in HFP; however, there are significant hardware and software differences between the devices that could cause variation and differences in the MPOD measurements obtained by both devices. To avoid any systematic bias in testing, a computerized randomized sequence was generated that accounted for the random selection of which device the participant used. The measurements of MPOD were performed in a randomly selected eye of the participant, and each participant performed the MPOD testing three times with both devices. Each MPOD measurement took approximately 2 to 2.5 min of time and at least a two-minute break was provided after a set of MPOD measurements. Participants performed MPOD measurements three times after instructions. The comparison of the first measurements obtained by the Zx Pro and the QuantifEye devices with their respective second and third measurements to assess the learning curve. If the values were statistically significantly different, a learning curve was considered to be significant. The difference between the second and the third measurements of the respective devices were used to assess repeatability and agreement.

### 2.2. QuantifEye MPS II

QuantifEye MPS II (QuantifEye IDE Vision Ltd., Caldicot, UK). was utilized as the predicate device to which the new device data were compared. Comprehensive details about the QuantifEye technology, its repeatability, and its agreement with other research prototypes can be obtained from elsewhere [26,27]. Participants were instructed and prepared to take the test as explained above.

### 2.3. Zx Pro—A New Handheld Heterochromatic Flicker Photometer

The new device, known as the Zx Pro (EyePromise/ZeaVision LLC, Chesterfield, MO, USA) is a device that measures MPOD and is based on the principles of HFP (explained above). The Zx Pro is a portable handheld device with audio prompts to guide the process of testing. The study participants hold and operate the device as per the audio prompts instructions. Its body includes a lower handheld portion and an eyepiece viewing tube, which is positioned transversely to the lower handheld portion (see schematic Appendix A). The handheld portion consists of a user input button, an LCD display for showing the MPOD score, and the battery and instrument electronics. The viewing tube terminates in an eye cup and includes the light sources. The device also has the ability to place a refractive lens to correct individual refractive errors.

### 2.4. Differences Between the MPOD Measuring Instruments

The Zx Pro is a portable handheld device that the individual participants hold in their hand and can use in a naturally seated position, whereas the QuantifEye is a desktop device that requires hunching over a table to view the target. The smaller handheld design changes the optical requirements, and the Zx Pro uses a 14-diopter lens compared to the 5-diopter lens used in the QuantifEye MPOD desktop device. To expedite the test, the Zx Pro proactively changes the starting frequency based on the user’s previous perceived frequency. This adaptive approach reduces the time required for the user to perceive the flicker, enhancing test efficiency. Prior to calculating the MPOD score, the Zx Pro performs a secondary check on the minimum perceived frequency. This additional verification step is designed to enhance the accuracy and reproducibility of the MPOD score and, in turn, shorten the learning curve.

### 2.5. Sample Size Estimation

Prior studies that have evaluated repeatability have utilized a sample size of 23 to 72 subjects [7,23,24,27,28]. For the correlation analysis, a two-tailed analysis setting the alpha and beta error at 1% and an expected correlation coefficient of 0.7, with a sample size of 28, was deemed sufficient to obtain statistical significance. The low alpha and beta error was selected to provide a decreased error of rejecting null hypothesis [29]. Further, to detect a significant difference of 0.02 du between measurements, an alpha error of 5%, and a power of 90%, a sample size of 58 was calculated as sufficient to detect significance [29].

Statistical analysis was performed using IBM SPSS 29. The differences between mean MPOD at various attempts practice, attempt 1 and 2 were assessed using repeated measures analysis of variance. The contrasts or differences between the groups were assessed using a paired samples *t*-test. The scatter plots, along with Pearson correlation coefficients, were performed to assess the correlations, and repeatability was assessed using Bland and Altman plots, and bias, along with 95% limits of agreement, were reported. Regression analysis was performed to assess the best goodness of fit model to predict Zx Pro MPOD from measured QuantifEye measurements. All graphical analyses were performed using the Analyze it for Microsoft Excel v2.30.

## 3. Results

The mean and standard deviation of all MPOD measurements are provided in the table (Table 1) below.

### 3.1. Repeatability of MPOD Measures QuantifEye MPS II

**Figure 1:**
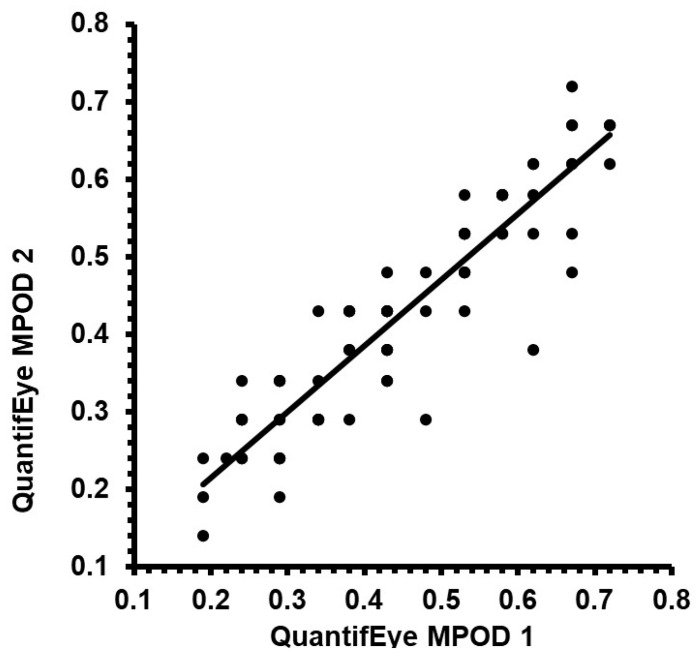
Scatter plot comparing first study-related measurement and second measurement obtained using the QuantifEye.

**Figure 2:**
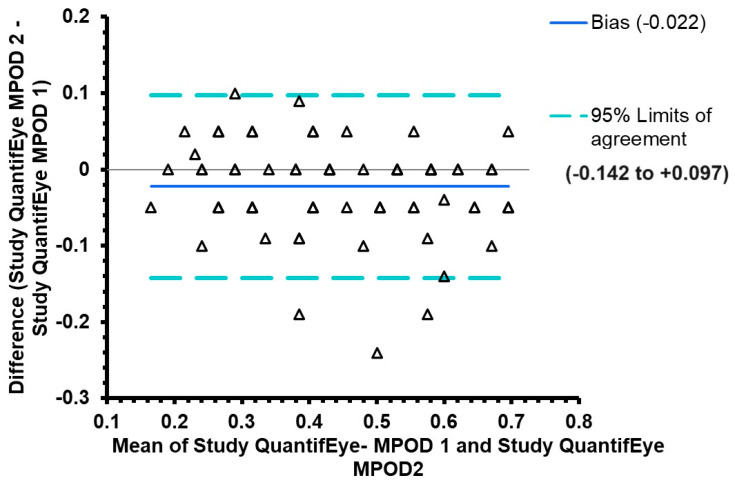
Bland and Altman plots comparing the first study-related measurement and second measurement obtained using the QuantifEye.

Figure 1 provides the Pearson correlation scatter plot for the MPOD measurements obtained using the two study measurements of QuantifEye. As expected, there was a positive linear correlation with minimal scatter representing physiological variability. The correlation coefficient between the study measurements of MPOD was excellent and statistically significant with an r-square of 0.846 (t-statistic 20.05, *p* < 0.0001) which signifies an 84.6% correlation between two measurements. Figure 2 provides the Bland and Altman plots of agreement between the first and second measurements, the 95% of the limits of the agreement being −0.142 to +0.098. There was a tendency for the second measurement to be higher than the first one at a lower MPOD score (t-statistic −3.18, *p* = 0.002).

### 3.2. Repeatability of MPOD Measures Using Zx Pro

**Figure 3:**
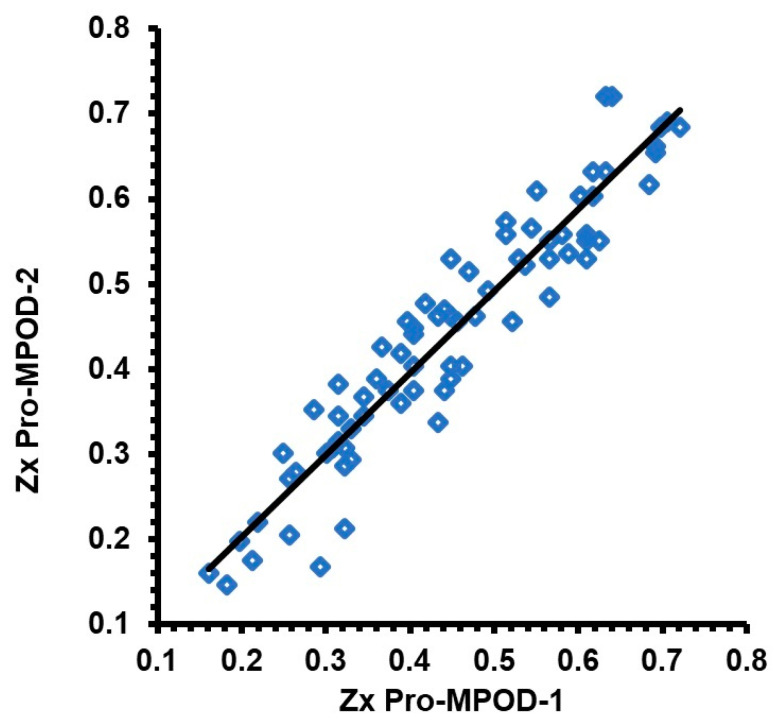
Scatter plot comparing first study-related measurement and second measurement obtained using the Zx Pro.

**Figure 4:**
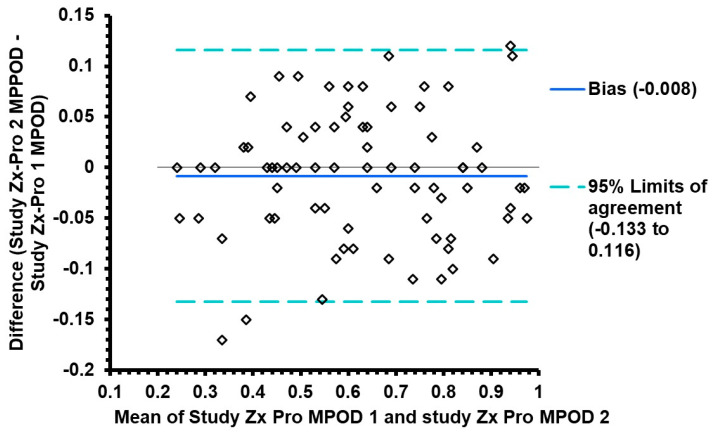
Bland and Altman plots comparing the first study-related measurement and second measurement obtained using the Zx Pro.

Figure 3 provides the Pearson correlation scatter plot for the MPOD measurements that were obtained using the second set of Zx Pro measurements. As expected, there was a linear correlation with minimal scatter representing physiological variability. The correlation coefficient between the first and the second MPOD measurements was excellent and statistically significant with an r-square 0.900 (t-statistic 25.87, *p* < 0.0001), which signifies a 90% correlation between the two measurements. Figure 4 provides the Bland and Altman agreement plots between the first and second measurements. The mean difference, as examined by bias analysis, was −0.008, which was not significant (t-statistic = −1.14, *p* = 0.26). The 95% limits of agreement were −0.133 to +0.116.

### 3.3. Comparison of Repeatability Between the QuantifEye and Zx Pro

Table 2 provides the descriptive statistics of the differences between the first and the second study measurements. Examining the difference range in first and second measurements obtained with both the devices, we find that the Zx Pro is marginally superior to the predicate device QuantifEye.

To further explore the difference in repeatability, we evaluated the coefficient of variation and the statistical significance between the difference in the first and second study measurements obtained using both devices. The coefficient of variation is calculated as the standard deviation of the difference in the first and second measurements divided by the means of measurement. The coefficient of variation between the first and second measurements of QuantifEye was 0.138, whereas the Zx Pro values were 0.104, indicating a relatively superior performance of the Zx Pro.

We performed a repeated measures ANOVA with contrasts to evaluate the learning curve in measured MPOD by different devices. As seen in Table 1, comparing the data obtained using the QuantifEye, we find that there is a small yet statistically significant difference between the practice attempt and the study-related measurements 1 and 2 (Repeated measures ANOVA *p* = 0.015). The paired samples *t*-test was performed to evaluate the differences between the groups. Although the difference in the practice attempts was not clinically significant when compared to the study measures, the mean difference between the two study-related measurements, 1 and 2, was 0.022 du, which reached statistical significance (paired samples *t*-test *p* values 0.21, 0.10 and 0.002, respectively). The mean MPOD obtained using the Zx Pro was not different when evaluated as a group (repeated measures ANOVA *p* = 0.325) and with paired comparisons (paired samples t- test *p* = 0.44, 0.20 and 0.26, respectively). This analysis indicates that there is a small yet statistically significant learning curve with QuantifEye, whereas the Zx Pro does not have a significant learning curve.

### 3.4. Agreement Between QuantifEye and Zx Pro MPOD Measures

**Figure 5:**
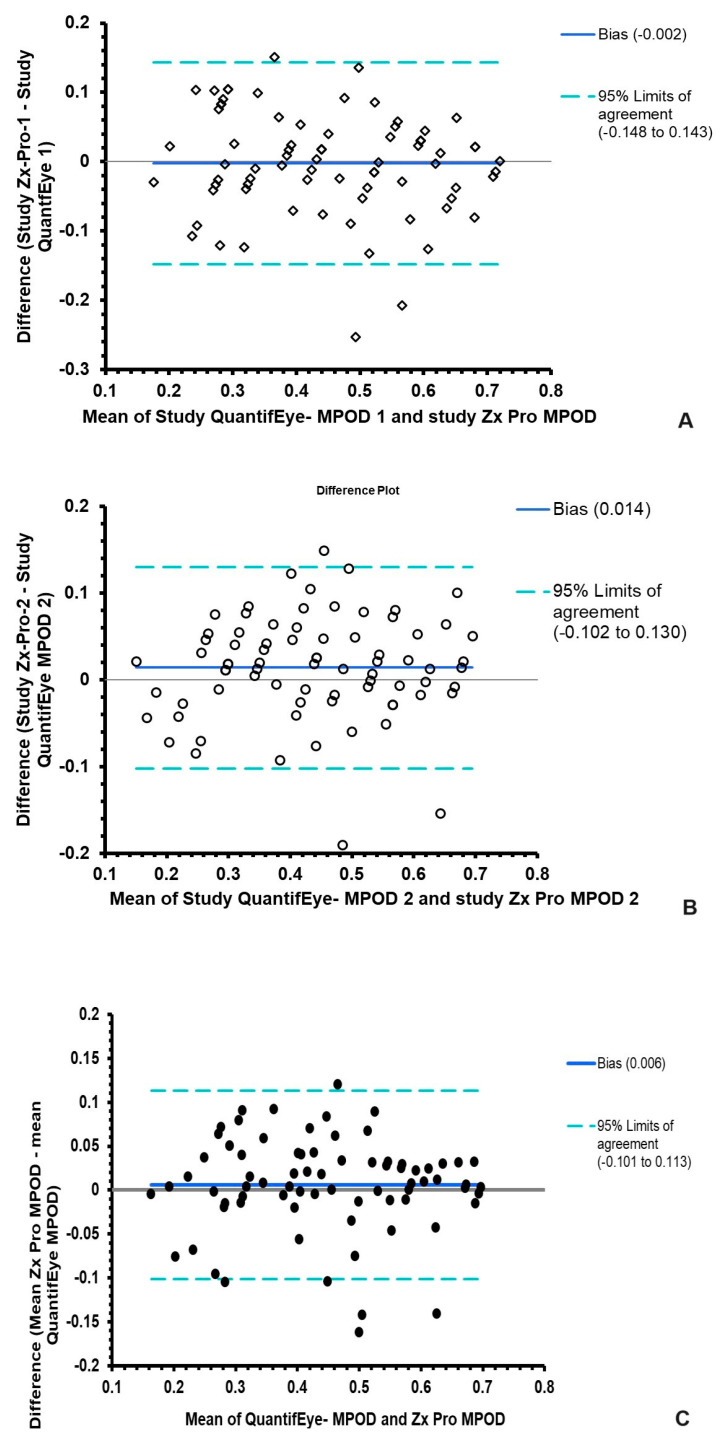
Bland and Altman Plots demonstrating agreement between the predicate device QuantifEye and the Zx Pro. (**A**) is the comparison of the first Study measurements; diamond symbol are data points, (**B**) is the comparison of the second study measurements; open circles are data points and (**C**) is the mean of the first and second measurements obtained with both devices, closed circles are data points.

The Bland and Altman analyses were performed to evaluate the agreement between the predicate device, QuantifEye, and the new device, Zx Pro. Table 3 provides the mean bias and 95% limits of agreement between the device’s first measurement, second measurement, and average data. The limits of agreement are narrow, and a bias value of close to zero in all three analyses indicates that there is no systematic bias between the devices (see Figure 5A–C)

### 3.5. Predicting Zx Pro MPOD Measures from QuantifEye Data

Given that the QuantifEye heterochromatic flicker photometry is clinically utilized, and numerous patients are followed up using the technology, it becomes essential to have a conversion factor to aid the clinical transition to new technology. We used regression analysis to analyze the scatter plots and the goodness of fit to evaluate the observed versus predicted values. We assessed a total of five regression models that included exponential, linear, logarithmic, polynomial, and power. Of the various models investigated, a linear regression analysis provided the best goodness of fit to the model. Using the linear regression analysis, one can expect to predict Zx Pro data with an accuracy of 86.4%.

Zx Pro MPOD = 0.915 × QuantifEye MPOD + 0.0434(1)

## 4. Discussion

Carotenoids are an essential part of the human diet, and xanthophylls that are concentrated in the retina and neural elements seem to have a much bigger role to play than previously recognized [1,9,10,11,12,14,15,16,17]. The xanthophyll carotenoids, lutein and zeaxanthin and its isomers, accumulate at different layers in the retina and in the fovea centralis, where a significant deposit is visually identifiable [1]. Measurement of this region provides the MPOD of a person’s eye, and the level of the MPOD is associated with and is an indicator of ocular health, systemic health, and disease states. MPOD is particularly interesting as it is a modifiable biomarker that can be increased with improved diet or nutritional supplementation [10,30,31]. The accurate and reliable measurement of such biomarkers is paramount, and prior-generation devices like the QuantifEye HFP have been shown to be repeatable and valid at measuring changes in MPOD over time [6,27,28,32,33].

Despite the seeming clinical acceptance of such technology, the widespread use of HFP devices has been limited due to various reasons. Some of the shortcomings of prior technology were the relatively larger footprint in the clinic, its lack of portability, and its ease of use. The QuantifEye is also significantly (five times) more expensive than the Zx Pro device. With these shortcomings in mind, the Zx Pro was introduced, which was portable, handheld, and convenient for individuals to perform the test in their natural seated position. The portable nature of the device allows it to be more mobile and move from one clinic room to another and perhaps may improve patient flow in busy clinics. However, it should be noted that handheld devices may not be preferred by individuals that have hand tremors and may need to support their hand on a table. This study evaluated the new technology Zx Pro, and found that the device provides MPOD measurements that are repeatable and in good agreement with the predicate device. The limits of agreement between Zx Pro and the predicate device QuantifEye are narrow, and no systematic bias was observed in the measurements.

Further, we found that the Zx Pro provides a shorter learning curve compared to the predicate device QuantifEye. The learning curve is an essential issue for all psychophysical tests. The mean difference between the practice attempt and the study-related measurements of QuantifEye MPOD was 0.022. Although such small differences may not be considered clinically significant, the learning curve is statistically significant. Compared to the predicate technology, QuantifEye, the Zx Pro provides data that are not significantly affected by such learning-related effects. Multiple reasons could explain the difference in the learning curve and the slightly superior reproducibility of Zx Pro. The portable handheld nature of the device allows for a more comfortable testing environment, and the software-related differences in identifying the endpoint of the tests may account for the superior performance of Zx Pro.

This study also provides a conversion equation that will allow clinicians to predict Zx Pro results using the QuantifEye measurements with an accuracy of 86.4%. It will be helpful for physicians that are used to seeing the QuantifEye MPOD to transition to Zx Pro and aid clinical decisions.

Additionally, we also evaluated repeatability by assessing the coefficient of variation. The coefficient of variation is a very important metric in assessing the clinical accuracy of a device. Our prior studies [27,28] that have evaluated the coefficient of variation in HFP devices have reported a value of 11–13% and other investigators have reported similar values [6,23,24,25,26]. We find that in this study the coefficient of variation in QuantifEye is around 14% which is comparable to the prior published literature. The Zx Pro has better repeatability when compared to the QuantifEye, with a coefficient of variation of 10%. This value is of paramount clinical utility as it can provide a clinically measurable endpoint. An increase of 10% in MPOD from baseline as measured by Zx Pro can be considered a “true” improvement and not a random function of “noise or chance”.

This study has some limitations. This study assessed the short-term repeatability by measuring MPOD multiple times in a short period of time. It would be ideal to evaluate long-term repeatability, that is, measurements 1–2 weeks apart. It should be noted that true long-term repeatability, perhaps months apart, cannot be assessed, as the various physiological situations like dietary intake, weight, and fat content changes, and oxidative state in the retina changes the level of carotenoids in the central fovea. Another possible limitation is that a single observer instructed all of the participants. The variability may be greater if multiple technicians were used to instruct the participants as individual technicians may vary in their education style.

It is known that, due to age-related absorption changes in the human crystalline lens, an error is introduced, which leads to an overestimation of MPOD in older adults [22,23,26,34,35]. Two methods have been utilized to overcome this error in measurements. Research laboratories use elaborate techniques of measuring central and peripheral measures, and the subtraction of two provides an internal control and accounting for the age-related yellowing of the lens [36,37,38]. The problem with this technique is that it is incredibly laborious, time-consuming, and prone to further errors in measurement. The second technique that clinicians can utilize is the built-in age-related correction of MPOD due to the age-related lens absorption changes. This technique is used in both QuantifEye and the Zx Pro devices and requires the age to input as a first step and the subsequent measurement of MPOD which yields better estimates of MPOD. This technique makes the MPOD measurements easier and shortens the testing time compared to the research laboratory techniques. For individuals who show a sudden decrease in visual acuity or exaggeration of cataracts, the MPOD measurements should be evaluated with caution, and, for the cases that had cataract extraction, the age should be permanently set to twenty-one.

Numerous studies have shown the importance of MPOD as a biomarker in health and disease states. Thus, it is important to discuss various methods of measuring the carotenoid status, particularly its direct or indirect relations with the MPOD levels. Carotenoids can also be measured using high-performance liquid chromatography (HPLC), which is considered the laboratory standard [39]. HPLC destroys the tissue and sample completely and cannot be performed in vivo in live patient eyes. HPLC can be performed to measure carotenoids in blood samples. This is a relatively expensive test and requires phlebotomy. Furthermore, the samples require specialized handling in laboratories, including storage in a dark environment and refrigeration at very low temperatures, usually negative 20 degrees Celsius. The measurement of blood levels of carotenoids tends to be more susceptible to recent dietary intake trends of carotenoids, is an indirect estimate of MPOD, and a weak correlation to an individual’s MPOD [39].

Total carotenoids can be objectively measured in the skin; these non-invasive and rapid tests are based on the principles of Raman spectroscopy [40,41,42] or Reflection spectroscopy [43,44,45], measure overall carotenoid levels of the skin, and may have a role in the assessment of carotenoids systemically. These devices have limitations and are susceptible to measurement-related issues like measuring consistently at the exact location measured previously and possibly to short-term variations like exposure to sunlight. Additionally, skin has numerous carotenoids and its metabolites [46], whereas the eye has only two dietary carotenoids, lutein and zeaxanthin, and a metabolite meso-zeaxanthin [3]. One would not expect the skin carotenoid composite scores to correlate well with MPOD, which is primarily zeaxanthin, meso-zeaxanthin, and lutein. Human skin biopsy studies and analyses with HPLC have shown lutein and zeaxanthin compromise around three percent of total carotenoids in the skin [47]. A recent study has shown that the skin carotenoid composite score by Refection Spectroscopy has a very weak correlation to MPOD (0.6%) and thus cannot predict the carotenoid status in the eye [48].

The HFP is a direct measurement of MPOD but a subjective technique that requires active input from the patient [6,7,14,15,16,27,28,49,50]. Although this technique appears simple and can be performed in a couple of minutes, it has the limitation of needing a response from the participants, which may be challenging to some. An ideal device would be one that can measure MPOD directly and easily and does not require any input or cooperation from the patients. Other research devices can measure MPOD either directly or indirectly, like autofluorescence techniques [51,52], Raman spectroscopy [53,54], or reflectometry [6,28,32]. These devices are standalone or require to be coupled with sophisticated equipment that is not commonly available. Some of these techniques are significantly affected by media opacities, possibly requiring the dilation of pupils [55,56,57]. Although such devices have been available in the research realms for about two decades, they have not become clinically available for various reasons. Cost plays a huge role in these devices not being available, costing 100–300 thousand US dollars. Currently, there are no objective techniques to measure MPOD that are commercially available and that the Food and Drug Administration has approved. The HFP has established itself as the clinical gold standard, and its lower cost, ease of use, and numerous validations from various research studies should see this technology make MPOD an essential biomarker in various general and specialty clinics.

## 5. Conclusions

This study evaluated the new portable heterochromatic flicker photometer, Zx Pro, and its ability to measure MPOD in a group of novice participants who ranged widely in age and refractive error. The results show that, despite the devices being subjective in nature and requiring active subject participation, they are capable of producing repeatable MPOD scores with a minimal learning curve and are in good agreement with the values obtained with a predicate device, QuantifEye.

## Figures and Tables

**Table 1:** MPOD measurements obtained using the QuantifEye and the Zx Pro.

Mean MPOD ^1^ Measurements	Practice Attempt	Study Attempt-1	Study Attempt-2	Repeated Measures ANOVA *p*-Value
QuantifEye	0.441 (0.16)	0.452 (0.16)	0.430 (0.14)	**0.015**
Zx Pro	0.459 (0.15)	0.447 (0.15)	0.444 (0.15)	0.325

^1^ MPOD stands for Macular pigment optical density, and measurements were performed in 76 eyes of 76 individuals. The values in parenthesis are standard deviations. Bold text *p* < 0.05.

**Table 2:** Descriptives of the difference in first and second measurements of the MPOD measuring devices.

Difference in First and Second Measurements	Mean	95% Confidence Intervals	Median	Minimum Maximum
QuantifEye	0.022	0.01 to 0.04	0.00	−0.10 0.24
Zx Pro	0.006	0.00 to 0.02	0.00	−0.08 0.12

**Table 3:** Agreement analysis.

Bland and Altman Analysis	The Mean Difference between QuantifEye and Zx Pro	95% Limits of Agreement
Study attempt-1	−0.006	−0.14 to +0.14
Study attempt-2 Mean of Attempts 1 and 2	+0.013 +0.006	−0.10 to +0.13 −0.101 to +0.113

**Figure A1:**
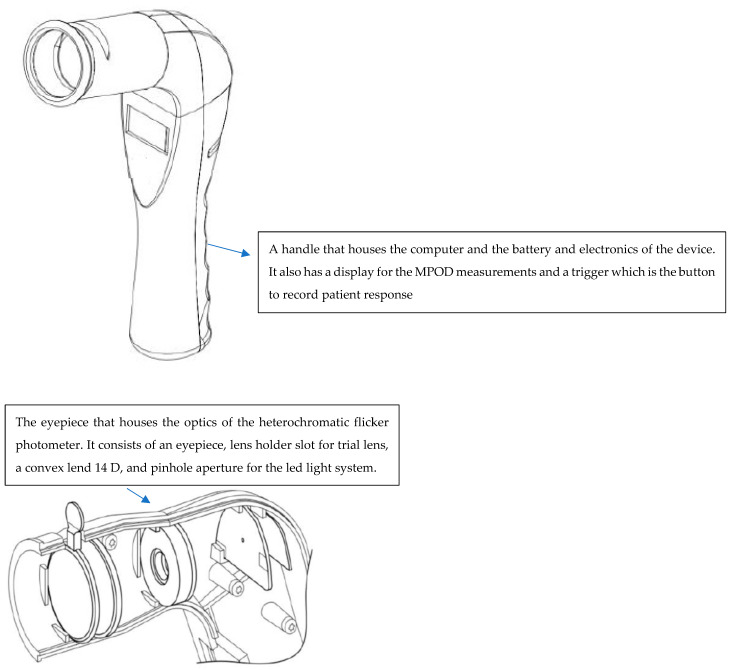
Schematic of the handheld heterochromatic flicker photometer Zx Pro.

## Data Availability

Available upon request after a data-sharing agreement is mutually acceptable.
